# Morphological Characteristics of Posterior Wall Fragments Associated with Acetabular Both-column Fracture

**DOI:** 10.1038/s41598-019-56838-5

**Published:** 2019-12-27

**Authors:** Siyu Tian, Yajie Chen, Yingchao Yin, Ruipeng Zhang, Zhiyong Hou, Yingze Zhang

**Affiliations:** 1 Department of Orthopaedic Surgery, the Third Hospital of Hebei Medical University, No. 139 Ziqiang Road, Qiaoxi District, Shijiazhuang, 050051 Hebei Province China; 2 Department of Hepatobiliary Surgery, the Third Hospital of Hebei Medical University, No. 139 Ziqiang Road, Qiaoxi District, Shijiazhuang, 050051 Hebei Province China

**Keywords:** Bone, Skeleton

## Abstract

Treatment of both-column fractures with posterior wall involvement is still a controversial topic. This type of posterior wall fracture is different from isolated acetabular posterior wall fracture (AO/OTA62-A1). The aim of this study is to compare the morphology of the posterior wall fragments of these two fracture patterns using computed tomography (CT) scans. All measured data were compared, and the differences between the groups (acetabular both-column fractures with posterior wall involvement were included in group A, and acetabular isolated posterior wall fractures were included in group B) were significant (P ≤ 0.05), including the direction angle, displacement, articular surface-posterior cortex ratio and articular surface area of the fracture fragment. The intraclass correlation coefficient of the measurements included inter-observer (ICC = 0.860) and intra-observer (ICC = 0.853). The morphology of the posterior wall fragments associated with both-column fractures is significantly different from that in isolated acetabular posterior wall fractures, and the treatment of the posterior wall fragment involved in both-column fractures of the acetabulum should be different from that of isolated acetabular posterior wall fractures.

## Introduction

**Figure 1:**
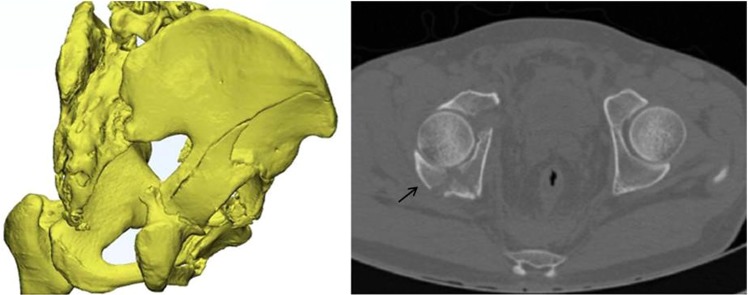
CT reconstruction and axial section images showing the posterior wall fragment associated with a both-column acetabular fracture.

In 1964, Judet *et al*. introduced a classification scheme for acetabular fractures that is still in use^1^. In this classification system, posterior wall fractures constitute the most common of the elementary patterns, including isolated and associated fractures (transverse-posterior wall and posterior column-posterior wall fractures)^2^. These types of posterior wall fractures are all caused by the impact of the femoral head on the posterior wall of the acetabulum posteriorly^3^. Previous studies in the literature have adequately reported the treatment and prognosis of these types of posterior wall fractures, suggesting that anatomical reduction and internal fixation are key to achieving good outcomes, unless the hip remains stable^4,5^. However, in our clinical work, we found that both-column acetabular fractures are sometimes associated with posterior wall fractures (Fig. 1), which is not in the Judet and Letournel classification system and represents 34.8% of cases, according to the literature^6^. In addition, the injury mechanism is the transmission of trauma medially to the anterior column, posterior column and quadrilateral plate. As a result, the posterior wall is pulled anteriorly by the hip joint capsule^5,7^.

The treatment of both-column fractures with posterior wall involvement is still a controversial topic, and the optimal method for the fixation of this type of posterior wall fracture is still unclear. As such posterior wall fractures are a complex type of acetabular fracture, most experts believe that they require additional rigid internal fixation. Some authors have suggested that combined simultaneous ilioinguinal and Kocher-Langenbeck approaches or extensile approaches are indispensable to obtain adequate visualization and anatomical reduction^8,9^. However, such approaches have been reported to have significant intraoperative and postoperative complications^10^. Wang *et al*.^7^ reported that this acetabular fracture pattern could be effectively managed by lag screw fixation of the posterior wall through a single ilioinguinal approach. Min *et al*.^11^ also reported that a single ilioinguinal approach could be used to treat such fractures and that whether the posterior wall should be fixed depends on the size and reduction of the fracture.

Due to the different mechanisms of injury, the posterior wall fracture fragment shape is quite different from that mentioned above. The purpose of this study was to compare posterior wall involvement in both-column fractures and isolated posterior wall fractures (AO/OTA 62-A1) of the acetabulum in terms of morphology and to find theoretical support for the treatment protocol of both-column acetabular fractures with posterior wall involvement.

## Results

The demographic details of the two groups were compared, and none of the differences in the measured variables were statistically significant (P > 0.05) (Table 1).

**Table 1:** Patient demographic characteristics.

Variable	BC + PW	Isolated PW	P value
Age(years)	46.95 ± 10.98	42.45 ± 9.58	0.175
**Gender**			
Male	17	16	1.000
Female	3	4	
**Affected side**			
Left	8	10	0.525
Right	12	10	
**Mechanism of injury**			
Fall	16	10	0.121
Vehicle accidents	4	8	
Others	0	2	

Regarding radiological measurements, the direction angles were 113.13° ± 7.98° (range: 96.39–127.62°) in group A and 58.35° ± 8.52° (range: 38.56–69.21°) in group B (P < 0.001). The medians of the displacement were 6.70 mm (interquartile range: 3.13 mm) in group A and 30.90 mm (interquartile range: 22.37 mm) in group B (P < 0.001). The means of the articular surface-posterior cortex ratio were 0.91 ± 0.13 (range: 0.72–1.16) in group A and 0.56 ± 0.08 (range: 0.44–0.70) in group B (P < 0.001). The articular surface areas of the two groups were 784.53 ± 246.53 mm^2^ (range: 374.00–1304.26 mm^2^) and 529.91 ± 256.05 mm^2^ (range: 225.01–1108.04 mm^2^), respectively (P = 0.005). The differences were statistically significant between the two groups (Table 2).

**Table 2:** Radiological characteristics.

Variable	BC + PW	Isolated PW	P value
Direction angle (°)			<0.001
Range	96.39–127.62	38.56–69.21	
Mean ± SD	111.13 ± 7.98	58.35 ± 8.52	
Displacement (mm)			<0.001
Median	6.70	30.90	
Quartile Range	3.13	22.37	
Articular surface-posterior cortex ratio			<0.001
Range	0.72–1.16	0.44–0.70	
Mean ± SD	0.91 ± 0.13	0.56 ± 0.08	
Articular surface area (mm^2^)			0.005
Range	374.00–1304.26	225.01–1108.04	
Mean ± SD	784.53 ± 246.53	529.91 ± 256.05	

The statistical analysis showed that all measurements had inter- and intra-observer ICCs higher than 0.80. The inter-observer ICC between observers I and II was 0.860, while the intra-observer ICC between the two measurements performed by observers I and II were 0.850 and 0.857, respectively (Table 3).

**Table 3:** Interobserver and intraobserver error of measurements: ICC.

Variable	Groups	ICC		
		Observer I	Observer II	I and II
Direction angle (°)	A	0.806	0.824	0.897
	B	0.817	0.886	0.802
Displacement (mm)	A	0.877	0.827	0.835
	B	0.914	0.867	0.933
Articular surface-posterior cortex ratio	A	0.841	0.918	0.846
	B	0.856	0.866	0.879
Articular surface area (mm^2^)	A	0.881	0.854	0.843
	B	0.806	0.813	0.842

Group A: Associated PW; Group B: Isolated PW.

## Discussion

The two types of posterior fractures have different mechanisms of injury. Judet and Letournel introduced the mechanism of acetabular fracture. Primary loading axes, including the femoral shaft, greater trochanter, and off-axis and the hip position, including the relative amounts of hip flexion, abduction/adduction, and internal/external rotation, determine the different types of acetabular fractures^1,12^. Both-column fractures with posterior wall involvement involve front stress fracture of the acetabulum; the trauma is a direct impact transmitted through the greater trochanter to the anterior medial wall of the acetabulum, which is divided into anterior and posterior column fragments when the hip is in external rotation and abduction^13^.The femoral head displaces medially, and the posterior wall fragment is pulled by the hip joint capsule anteriorly and is often a large-sized fragment, nondisplaced or minimally displaced, while the joint capsule is intact^7^.The mechanism of acetabular isolated posterior wall fracture involves the femoral head striking the posterior acetabular wall with the hip flexed. The fracture fragment is often comminuted and displaced visibly, and this fracture pattern involves posterior hip dislocation^2,3^.

The disparate mechanisms cause obviously different morphological characteristics, which result in different displacements between the two groups. The displacement of the fracture fragment in group A (median: 6.70 mm, interquartile range: 3.13 mm) was significantly smaller than the displacement in group B (median: 30.90 mm, interquartile range: 22.37 mm), indicating that the damage of the posterior acetabular surrounding tissue in group A was less than that in group B. The displacement directions of the posterior wall fractures in the two fracture patterns were also different. Tosounidis *et al*. reported that the posterior wall fragment, which is associated with both-column fracture, is created by a “pull-type” mechanism^5^ and has a tendency to move anteriorly. Moed *et al*. reported that the hip joint capsule and surrounding soft tissues may contribute to the overall stability of the hip^14^. In addition, Vailas *et al*. conducted a cadaveric study and reported that the joint capsule plays an important role in the stability of the hip^15^. Therefore, the integrity of the capsule plays an important role in the hip stability of acetabular both-column fractures with less displacement of posterior wall involvement.

The posterior wall fragment in isolated posterior wall fractures (AO/OTA 62-A1) of the acetabulum was moved posteriorly directly by the femoral head^1,16^. Although closed reduction of the hip posterior dislocation was undertaken, the fragment usually could not be reduced. Although it was sometimes reduced after correction of the hip posterior dislocation, the fragment still had an obvious tendency to move posteriorly, and the remaining posterior wall was not large enough to maintain the femoral head within the acetabulum, which resulted in instability of the hip, and a Kocher-Langenbeck approach was required for its correction^2,3,14,17^. Some authors found, using 2D CT, that even a few posterior wall fragments with less than 20% of wall involvement may be unstable^18,19^, and others have suggested that the standard criterion is a remaining posterior wall with a size large enough to maintain the femoral head within the acetabulum^14^.Three methods have been applied to measure the deficit of the posterior wall using CT, as described by Calkins *et al*., Keith *et al*., and Moed *et al*., and fragment sizes measuring greater than 65.7%, 40%, and 50%, respectively, were deemed to be unstable^14,20,21^. However, all of the above studies examined isolated posterior wall fractures of the acetabulum. Stability studies evaluating acetabular both-column fractures with posterior wall involvement have rarely been reported.

Wang *et al*.^7^ found that fracture patterns of posterior wall fragments of both-column fractures were different from the posterior wall patterns of other types of fractures, including transverse and posterior wall or posterior column and posterior wall fractures, due to differences in the mechanism of injury. For such fracture patterns of the acetabulum, preoperative CT scans were used to measure the variables (height, relative depth, displacement and peripheral length, which were 27.8 ± 2.5 mm, 71.5 ± 5.3%, 5.0 ± 3.2, and 23.0 ± 2.3 mm, respectively) in place of the previous methods, and fracture fragments were considered large in size and minimally displaced according to the measurement results. The methods were similar to those of our study for certain variables, but the morphologies and measurement results of the two types of fragments were not compared.

The differences between the two types of fracture in the other three measured parameters, including the direction angle, articular surface-posterior cortex ratio and articular surface area of the fracture fragment, were statistically significant. The above three measurement indexes reflected the difference of the fracture lines of the two types of posterior wall fractures from different 2D images. The fracture of the posterior wall fragment in group A often had a large and deep articular surface area, and the direction angle was an acute angle (mean ± SD: 58.35° ± 8.52°, range: 38.56–69.21°) in axial CT sections, which indicated that the fracture line had an oblique orientation that extended from the anterior to the posterolateral fragment of the posterior wall. For this type of acetabular fracture, when the iliac wing was fractured, the iliopubic and ischiadic fragments were reduced and fixed successively, and the fixed posterior column and residual posterior wall were the posterior mainstays of the posterior wall fragment. In other words, the fixed posterior column and residual posterior wall acted as a barrier against the posterior wall fragment moving posteriorly, if possible. In contrast, the fracture of the posterior wall fragment in group B often had a small and narrow articular surface area, and the fragment length of the posterior cortex was longer than the articular internal length in axial CT sections. The direction angle was obtuse (mean ± SD: 113.13° ± 7.98°, range: 96.39–127.62°), which showed that the orientation of the fracture line extended from the anterior to the posteromedial fragment of the posterior wall and was opposite to the previous fracture line of group A. Therefore, no block or support existed for the acetabulum toward the posterior wall fragment in the rear if it displaced posteriorly.

In addition, the reliability of the measurements obtained in this study was assessed using ICC values, with values higher than 0.75 considered strong agreement and correlation. The ICCs of all inter- and intra-observer measurements were higher than 0.80, indicating that the method showed a high level of reproducibility and reliability.

This study had several limitations. It was a retrospective review with a relatively small sample size drawn from a database because of the relatively low incidence of both-column fractures with posterior wall involvement. Additional cases should be evaluated to verify the characteristics of such posterior wall fractures. Second, although some measures, including using the mean value of selected, multiple, typical sections and reversed copy matching on the uninjured, contralateral hip, were applied to minimize the error, interference factors from the surveyor still exist, which requires further improvement. Third, there is systematic error in the measurement of the displacement of the posterior wall, that is, it is only measured from the two-dimensional CT. Even after the body position correction, it is difficult to accurately find the corresponding measurement point on the posterior wall when the fragments are severely comminuted. Another limitation of this work was that the stability of such fracture patterns was only analyzed theoretically in terms of anatomical morphology and not clinical outcome. In future clinical practice, a prospective randomized controlled trial, including radiographic results, clinical outcomes and postoperative complications, will be conducted to confirm whether it is required to fix posterior wall fragments associated with acetabular both-column fractures.

## Methods

### Ethics statement

This study has been reviewed and approved by the Institutional Review Board of the Third Hospital of Hebei Medical University, and all aspects of the study comply with the Declaration of Helsinki. Informed consent was obtained from all patients in the study.

### Patients

This was a retrospective study of patients with acetabular fractures consecutively treated by open reduction internal fixation (ORIF), and all cases were examined at a level I trauma center between February 2015 and April 2018. The inclusion criteria were patients who were older than 18 years, had achieved musculoskeletal system maturity and had a unilateral traumatic acetabular fracture or acute fractures (<21 days). The exclusion criteria were pathological fractures, bilateral acetabular injuries, preexisting ipsilateral hip diseases, or femoral head fracture. The patients were divided into two groups: group A (Associated PW) consisting of 20 patients who presented with acetabular both-column fractures with posterior wall involvement; and group B (Isolated PW), consisting of 20 patients with acetabular isolated posterior wall fractures. All patients were placed in a supine position to undergo preoperative pelvic computed tomography performed using a SOMATOM Sensation 64 CT scanner (Siemens, Erlangen, Germany) at a 1.0 mm slice thickness. The obtained CT scans were assessed in terms of the fracture pattern by two experienced orthopedists according to the Judet and Letournel classification system. Patient demographics and characteristics are shown in Table 1.

### Radiological measurements

CT scan digital imaging communication in medicine (DICOM) imaging files were used as input data for the Mimics 20.0 program (Materialise Inc., Belgium) to measure and compare the characteristics of posterior wall fragments between the two groups.

The body position was corrected to the standard supine position using the Reslice function before measurement. The posterior wall fragment measurements were performed using the largest amount of fractured posterior wall (i.e., the smallest intact remaining posterior acetabulum) by selecting all axial computed tomographic sections^14,19^. To minimize errors, three consecutive typical sections were measured, and the mean value was recorded. The measurement variables and techniques were as follows.

**Figure 2:**
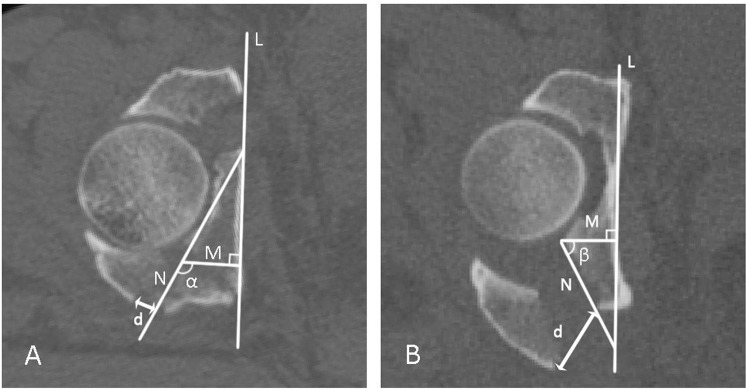
Measurement of the direction angle and displacement of group (**A**) (Associated PW) and group (**B**) (Isolated PW).

Direction angle of the posterior wall fracture: At the level of the largest posterior wall deficit in group A, a straight line (L) was drawn as close as possible along the cortex of the medial wall of the injured acetabulum (in group B, the line was drawn directly along the medial wall of the injured acetabulum, while in group A, the line was drawn along the contralateral medial wall symmetrical to the line on the injured side). Next, a line (N) was drawn along the fracture line of the residual posterior wall of the acetabulum. Then, a line (M) was drawn through the any point on line N perpendicular to line L. The angle (α) formed by lines M and N was the direction angle of group A (i.e., the angle between line M and the fracture line of the posterior wall)^22,23^. The same method was used to measure the direction angle (β) of group B (Fig. 2).

The displacement of the posterior wall fracture was the distance from the lowest point of the residual posterior wall of the acetabulum to the corresponding point on the posterior wall fracture fragment, and the distance (d) was defined as the displacement of the two groups (Fig. 2).

**Figure 3:**
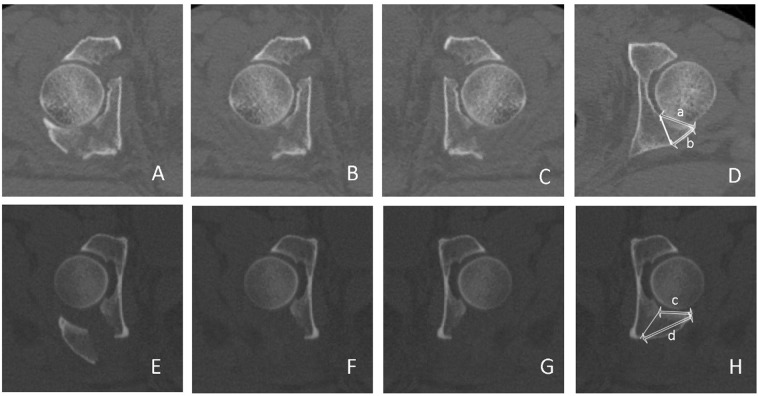
Articular surface-posterior cortex ratios of group (**A**) (Associated PW) and group (**B**) (Isolated PW). (**A**,**E**) Axial CT sections of the injured acetabulum; (**B**,**F**) Injured acetabulum without the fracture fragment of the posterior wall. (**C**,**G**) The reversed copies from (**B**,**F**), respectively. (**D**,**H**) Selected CT sections matching the uninjured acetabulum and simulating a fracture line. The lengths (a–d) were measured between the internal and posterior cortex, respectively.

When measuring the articular surface-posterior cortex ratio, the patients’ positions were usually not exactly perpendicular to the gantry in the CT scanner, and the fracture fragment was displaced after the injury. To minimize errors, a “reversed copy” of the uninjured, contralateral hip was used to obtain an exact match by comparing femoral head sizes and the configuration of the acetabulum^14^. The results of the two groups were calculated from the ratio of the measured length of the posterior cortex to the articular internal length (a/b and c/d) (Fig. 3).

**Figure 4:**
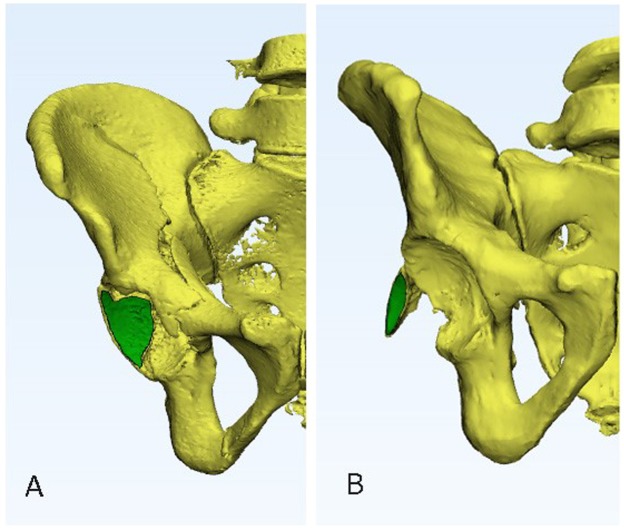
CT reconstruction of an image used to calculate the articular surface areas of group (**A**) (Associated PW) and group (**B**) (Isolated PW).

Articular surface area of the fracture fragment: The injured femoral head was removed on the CT reconstruction in Mimics 20.0. The 3D reconstruction was then converted by 3-Matic 12.0, and the articular surface area of the fracture fragment was measured (Fig. 4).

All measurements were performed by two orthopedists at intervals of least two weeks and evaluated for inter- and intra-observer consistency.

### Statistical analysis

All statistical analyses were performed using SPSS version 13.0 (SPSS Inc., Chicago, Illinois). Descriptive statistics are presented as the mean ± standard deviation or median and interquartile range. Displacement was analyzed in the two groups by Mann-Whitney U tests. Other radiological measurements were analyzed by independent-samples T tests. Comparisons of demographic details between the two groups were conducted using chi-squared tests. A value of P ≤ 0.05 was considered statistically significant. Inter-observer and intra-observer reliability were assessed by using the interclass correlation coefficient (ICC), with a value higher than 0.75 considered strong reliability^24^.

## Conclusion

The extent and direction of the displacement, radiological characteristics of the fracture line and fracture fragment morphology of the posterior wall associated with acetabular both-column fractures were different from those of acetabular isolated posterior wall fractures, which revealed significant differences in the mechanisms of injury and post-acetabular stability between the two types of fracture. Treatment of the posterior wall fragment involved in both-column fractures of the acetabulum should be different from that for the isolated acetabular posterior wall fracture.
